# The impact of physical exercise on the flourishing level of vocational college students: an analysis based on random forest regression algorithm and moderated mediation model

**DOI:** 10.3389/fpubh.2026.1778344

**Published:** 2026-04-24

**Authors:** Fan-Zheng Mu, Ming-Jia Li, Li-Han Lin, Rui Chen, Zan Huang, Hu Lou, Sai Zhu, Jian-hua Xu

**Affiliations:** 1 School of Physical Education and Sport Science, Fujian Normal University, Fuzhou, Fujian, China; 2 China Basketball College, Beijing Sport University, Beijing, China; 3 School of Sports Science, Nantong University, Nantong, China; 4 Student Affairs Office, Nantong Health Higher Vocational and Technical College, Nantong, China

**Keywords:** flourishing levels, moderated chain mediation model, physical exercise, random forest regression algorithm, vocational college students

## Abstract

This study examined the associations among physical exercise, sensory processing sensitivity, depression–happiness, relational energy, and flourishing in vocational college students, with a focus on the serial mediating roles of sensory processing sensitivity and depression–happiness and the moderating role of relational energy. Using a multistage sampling design, 11,388 vocational college students from mainland China were surveyed. Physical exercise did not show a significant direct positive association with flourishing, but did so indirectly through sensory processing sensitivity and depression–happiness. Relational energy significantly moderated the association between depression, happiness, and flourishing. After controlling for gender, grade, and BMI, physical exercise showed a significant negative direct association with flourishing. These findings suggest that the benefits of physical exercise for flourishing operate primarily through personality, emotional, and interpersonal pathways.

## Background

The National Report on Mental Health Development in China shows that 41.1% of male university students and 46.6% of female university students are at some level of anxiety risk, while 20% of vocational college students are at mild to moderate risk of depression (1). This raises an important question: despite strong national efforts to promote physical exercise, such as the National Physical Fitness Standards, why do vocational college students remain vulnerable to psychological and behavioral problems? Therefore, it is essential to explore the factors that prevent them from achieving optimal mental health.

“Flourishing” represents a higher level of mental health and reflects the optimal positive functioning of an individual's psychological capacities (2). Individuals with higher flourishing tend to show better life satisfaction, a stronger sense of belonging, and greater emotional regulation ability (3). According to Flow Theory, when students engage in activities that match their skills to challenges, they are more likely to enter a state of deep concentration and immersion (4). As a mind-body activity, physical exercise may not only alleviate negative emotions such as depression, but also promote positive mental health, including flourishing. However, its effects are not simply linear; rather, they are shaped by multiple mediating and moderating factors, among which flow experience, self-efficacy, and social support are especially important (5, 6). At the same time, vocational college students' psychosocial background, individual traits, cognitive patterns, and social prejudice related to vocational education may influence whether these positive effects are reflected in improved flourishing. Existing studies have paid limited attention to the relationship between physical exercise and flourishing in the vocational college context. Therefore, this study proposes Hypothesis *H1: physical exercise is significantly correlated with the level of flourishing among vocational college students*.

Sensory Processing Sensitivity (SPS), also known as environmental sensitivity, is a temperament trait related to how individuals perceive and process sensory information (7). Research suggests that SPS can be divided into high, medium, and low levels, with high-sensitivity individuals accounting for about 30% of the population (8). Studies have shown that differences in physical activity preferences are associated with attachment patterns and levels of sensory processing sensitivity (9). From a behavioral perspective, individuals with higher SPS tend to prefer low-intensity, controllable activities to avoid over-arousal. When activity demands match their abilities, they are more likely to experience flow, which can promote positive emotions and self-realization. From a cognitive perspective, high sensitivity may enhance the processing of bodily, emotional, and environmental cues (10). In supportive contexts, this can help individuals better integrate the positive experiences gained from exercise, thereby enhancing flourishing; in stressful contexts, however, it may also increase attention to negative cues and rumination, which can hinder flourishing. Based on this, the present study proposes Hypothesis *H2: sensory processing sensitivity mediates the relationship between physical exercise and flourishing among vocational college students*.

Depression and happiness are two core but relatively independent dimensions of mental health, and low depression does not necessarily mean high wellbeing (11–14). The Dual-Factor Model of Mental Health emphasizes that true mental health includes both the absence of negative symptoms and the presence of positive psychological functioning, such as subjective wellbeing and flourishing (15, 16). Based on this framework, individuals can be classified into four groups, including completely mentally healthy, susceptible, symptomatic but satisfied, and mentally unwell (17). Previous studies using latent profile analysis have identified clear heterogeneity in the mental health status of Chinese university and vocational college students, highlighting the need for targeted interventions (18, 19). Existing evidence also suggests that physical exercise may not reduce depression directly, but may improve wellbeing by enhancing psychological resources and reducing distress (20–23). Based on this, the present study proposes Hypothesis *H3: depression-Happiness mediates the relationship between physical exercise and flourishing among vocational college students*.

Relational energy is a key concept in positive organizational behavior and resource conservation theory, referring to the psychological vitality and positive energy individuals gain through social interactions (4, 24–26). As an important personal resource, it can enhance vocational college students' learning engagement, subjective vitality, creativity, and positive emotions, while also helping reduce stress, emotional exhaustion, and academic burnout (27–31). Because flourishing includes dimensions such as positive emotion, meaning in life, and interpersonal relationships, relational energy may play an important role in promoting flourishing through a social-emotional pathway. In particular, higher relational energy may both directly support flourishing and buffer the negative influence of poorer depression-happiness states. Based on this, Hypothesis *H4 is proposed: relational energy moderates the relationship between depression-happiness and flourishing among vocational college students, such that stronger relational energy weakens the impact of depression-happiness on flourishing*.

In summary, this study seeks to test a moderated chain mediation model linking physical exercise, sensory processing sensitivity, depression-happiness, and flourishing among vocational college students, with relational energy acting as a moderator in the final stage. The study's theoretical value lies in clarifying how behavioral, personal, emotional, and social factors jointly relate to flourishing. Its practical aim is to provide an integrated evidence-based framework for promoting the mental health of vocational college students.

## Materials and methods

### Data sources

Data collection was carried out on-site by the research team across several higher vocational colleges in mainland China. A multi-stage sampling design was used to recruit students from different provinces to enhance the sample's geographic and socioeconomic representativeness. Following formal approval from the relevant institutional authorities, the questionnaire was administered online during students' midday breaks. Before participation, the Informed Consent Form was read aloud, and all students were required to confirm that they understood the study procedures before providing written informed consent. In total, 11,605 questionnaires were distributed, and 11,398 were returned. After data screening, including the removal of questionnaires with logical inconsistencies, omissions, errors, or unidentifiable responses, 11,388 valid cases were included in the final analysis. Data were collected between September and October 2025.

### Data screening and quality control

The following exclusion criteria were applied to ensure data validity: (1) Respondents younger than 18 or older than 23 years. (2) Incomplete questionnaires or those with missing responses. (3) Self-contradictory or logically inconsistent answers. (4) Questionnaires with identical responses across all items. (5) Data showing no variability.

### Ethical safeguards during data collection

Ethical safeguards were strictly implemented throughout the data collection process. Before the survey, our research team contacted the responsible staff at each participating institution, and access to the campuses was coordinated with the assistance of class counselors. At the survey site, the research team provided a standardized explanation of the questionnaire instructions and read the informed consent form aloud to all participants. Participation was entirely voluntary, and all students signed a paper-based informed consent form before completing the questionnaire. Confidentiality and anonymity were fully assured, and all responses were kept strictly confidential. In addition, students were informed that they could discontinue participation at any time without penalty. If a student experienced discomfort or resistance during completion and chose to stop midway, that questionnaire was excluded from the final analysis.

## Applied measures

### Physical Activity Level Scale (PARS-3)

Physical Activity Rating Scale (PARS-3): Physical activity was assessed using the revised PARS-3 (32). Intensity and frequency were rated from 1 to 5, and duration from 1 to 5 (scored 0–4). Total scores ranged from 0 to 100, with ≤ 19 indicating low activity, 20–42 moderate activity, and ≥43 high activity. Previous research reports a test-retest reliability of 0.82; in this study, Cronbach's α was acceptable (33).

### Highly Sensitive Person Scale (HSPS)

Highly Sensitive Person Scale (HSPS): Sensory processing sensitivity was measured using the HSPS, comprising three dimensions: ease of irritability, aesthetic sensitivity, and low sensory threshold (34). The Chinese version includes 14 items rated on a 7-point Likert scale. Prior validation studies demonstrate strong reliability and validity. In this study, Cronbach's α was 0.904 (7).

### Short Depression-Happiness Scale–Chinese Version (SDHS-C)

Short Depression-Happiness Scale–Chinese Version (SDHS-C): This six-item scale assesses depressive (Items 1, 3, 6) and wellbeing experiences (Items 2, 4, 5). Responses ranged from 1 (never) to 4 (often). Positively worded items were reverse-coded, and total scores reflected overall depression-happiness, with higher scores indicating poorer mental health. Cronbach's α in this study was 0.814. The scale was treated as a single composite variable in the moderated serial mediation model (13).

### Flourishing Scale (FS)

Flourishing Scale (FS): Flourishing was measured using the eight-item FS, rated on a 1–7 scale (35). Higher scores indicate greater flourishing. The Chinese version demonstrates strong psychometric properties. In this study, Cronbach's α was 0.841(36).

### Relational Energy Scale (RES)

Owens et al. (37) defined relational energy as the energy derived from interacting with others in social development, stimulating personal behavior. Relational energy was assessed using the five-item RES, rated on a 5-point Likert scale. Higher scores reflect higher perceived relational energy. Previous studies report strong reliability; Cronbach's α in this study was 0.832 (38).

## Statistical methods

Data analysis was conducted using SPSS 26.0, JASP 0.95, and Mplus 8.3. After screening, valid data were retained for analysis. Common method bias and variable correlations were examined. Random forest regression was used for exploratory analysis, followed by testing of a moderated serial mediation model with 95% confidence intervals (39–41).

## Analysis models and tests for fitness

In this study, a MIMIC model (Multiple Indicators Multiple Causes) was used. MIMIC is a special form of structural equation modeling that incorporates elements of the random forest algorithm. The model fit indices were as follows: CFI = 0.826, TLI = 0.744, NFI = 0.825, IFI = 0.827, and RMSEA = 0.096(<0.1). The overall model fit was relatively weak, but still acceptable (42–44). The fit indices of the moderated chain mediation model were as follows: χ^2^/df = 25.1, RMSEA = 0.046(<0.05), CFI = 0.991(>0.90), TLI = 0.948(>0.90), and SRMR = 0.012(<0.08), indicating that the model showed a good overall fit to the data.

## Ethical considerations

This study was conducted in accordance with the ethical principles of the Declaration of Helsinki and relevant national regulations on research involving human participants. Ethical approval and institutional permission were obtained from the relevant participating institutions before data collection. Specifically, approval/consent records included the Medical Ethics Review of Nantong University (Tong Da Lun Shen (2022) No. 70), the Ethical Review Report of Fujian Normal University (Approval No. FNU-L2025005), and the Ethics Review Report of Jiangsu Province Nantong Health Vocational and Technical School [Tong Weilun Shen (2024) No. 01].

## Common method bias test

To control for common method bias, this study strictly controlled the questionnaire design and administration process, such as using reverse scoring for some items and emphasizing strict confidentiality. Harman's single-factor test was used to analyze the presence of common method bias. The results indicated that five factors with eigenvalues greater than 1 were extracted, with the first factor explaining 35.118% of the total variance, which is below the 40% critical value threshold. This suggests that common method bias is unlikely to be a serious issue in this study (41). Additionally, this study employed the CFA marker variable method. Existing research recommends that marker variables should not show significant correlations with any other variables used in the study. Using AMOS software, the model without common method bias was compared to the model with common method bias. The results showed that after adding the common method bias latent variable, the fit indices improved, with the changes in CFI and TLI not exceeding 0.1, and the reductions in RMSEA and RMR not exceeding 0.05. This indicates that there is no significant common method bias in this study (as shown in Table 1).

**Table 1:** Comparison of confirmatory factor analysis fit indices for physical exercise, sensory processing sensitivity, depression-happiness, relational energy, and flourishing among vocational college students in China.

Model	RMSEA	RMR	CFI	TLI	IFI	GFI
Model without common method bias	0.088	0.387	0.843	0.83	0.843	0.815
Model with common method bias	0.083	0.349	0.86	0.848	0.86	0.839

## Results

### Descriptive statistical analysis

The results showed significant gender and grade differences in physical activity, BMI, and sensory processing sensitivity. Males and first-year students were at greater risk of low physical activity, whereas females showed higher sensory processing sensitivity and were more likely to report moderate-intensity activity. Overweight was also more common among females and first-year student (as shown in Tables 2, 3).

**Table 2:** Sample distribution of vocational college students in China by demographic characteristics.

Variable	Option	Frequency	Percentage (%)
Gender	Male	9,277	81.5
	Female	2,111	18.5
Year	Freshman	3,116	27.4
	Sophomore	2,742	24.1
	Junior	5,530	48.6
Activity level	Low activity	9,444	82.9
	Moderate activity	1,210	10.6
	High activity	734	6.4
BMI index	Normal	4,250	37.3
	Overweight	6,619	58.1
	Obese	519	4.6

**Table 3:** Descriptive statistics for physical exercise and sensory processing sensitivity among vocational college students in China.

Variable	Evaluation level	Male		Female		Freshman		Sophomore		Junior	
		*n = 9,277*	*%*	*n = 2,111*	*%*	*n = 3,116*	*%*	*n = 2,742*	*%*	*n = 5,530*	*%*
Physical activity level	Low physical activity	8,439	91	1,005	47.6	3,041	97.6	2,074	75.6	4,329	78.3
	Moderate physical activity	262	2.8	948	44.9	9	0.3	658	24	543	9.8
	High physical activity	576	6.2	158	7.5	66	2.1	10	0.4	658	11.9
	*χ^2^*	3260.569				1393.899					
	*p*	<0.001				<0.001					
	*Gramer's V*	0.535				0.247					
BMI level	Normal	4,033	43.5	217	10.3	485	15.6	1,466	53.5	2,299	41.5
	Overweight	4,727	51	1,892	89.6	2,565	82.3	1,266	46.2	2,788	50.4
	Obese	517	5.5	2	0.1	66	2.1	10	0.3	443	8.1
	*χ^2^*	1,063.429				1,342.361					
	*p*	<0.001				<0.001					
	*Gramer's V*	0.306				0.243					
**Variable**		**Male**		**Female**		**Freshman**		**Sophomore**		**Junior**	
		***M***	***sd***	***M***	***sd***	***M***	***sd***	***M***	***sd***	***M***	***sd***
Sensory processing sensitivity	Irritability	13.35	3.006	14.82	2.98	13.38	2.938	13.53	2.993	13.8	3.137
	Aesthetic sensitivity	17.72	3.506	19.12	3.456	17.71	3.548	17.95	3.428	18.15	3.578
	Low sensory threshold	18.4	3.312	19.58	3.203	18.44	3.355	18.58	3.237	18.74	3.345
	Total sensory sensitivity	49.47	8.982	53.52	8.841	49.53	8.979	50.06	8.811	50.69	9.268
	*η^2^*	0.0299855				0.00294845					
	*F*	351.969				16.834					
	*p*	<0.001				<0.001					

### Correlation test

**Figure 1:**
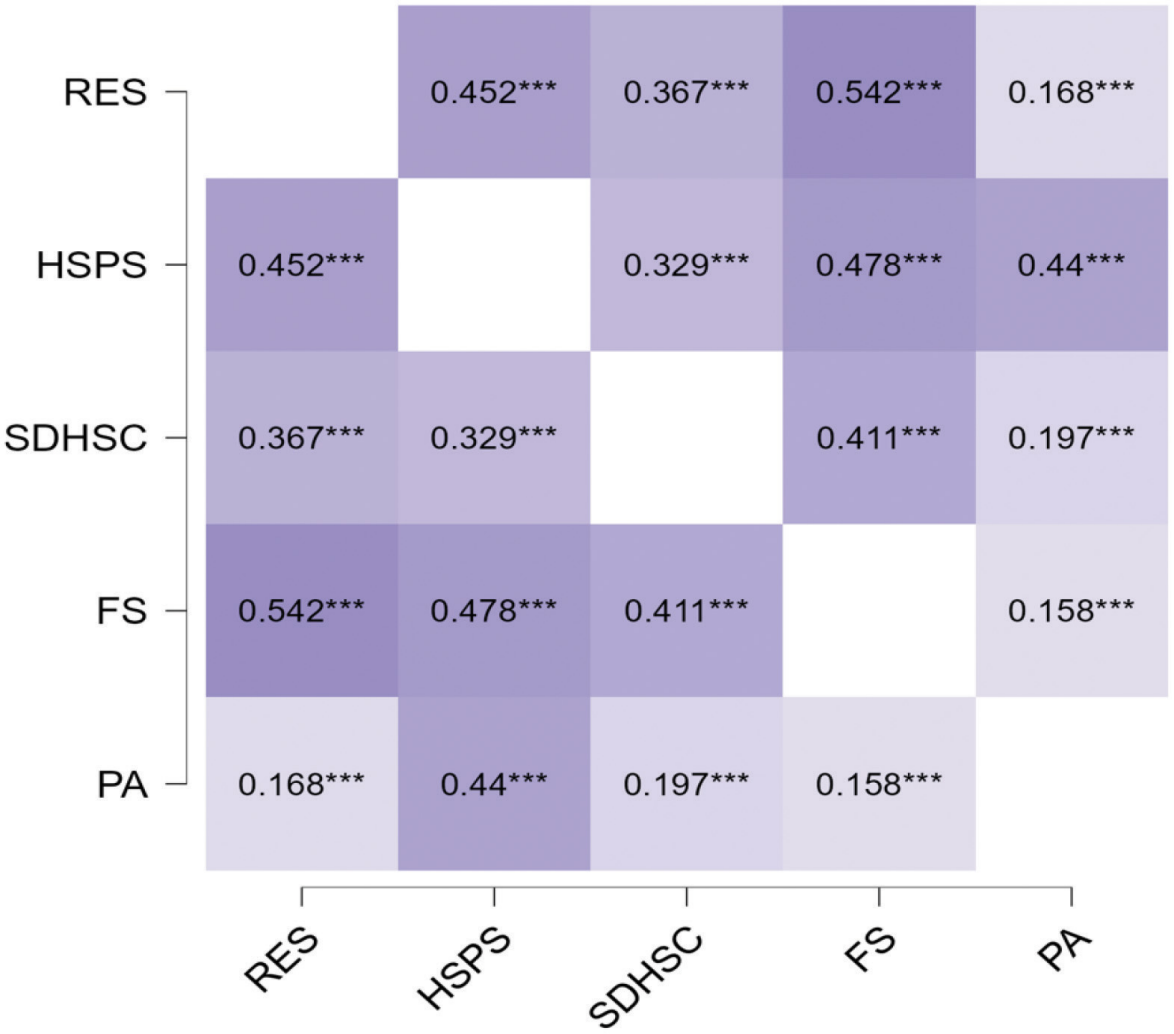
Correlation heatmap of physical exercise, sensory processing sensitivity, depression-happiness, relational energy, and flourishing among vocational college students in China. ^***^P < 0.001.

Physical exercise was positively associated with all study variables, with the strongest correlation observed for sensory processing sensitivity, supporting H1. Sensory processing sensitivity emerged as the most central variable, showing strong associations with flourishing, depression-happiness, and relational energy. Flourishing was also strongly related to relational energy, and Bayesian factors supported the robustness of these findings (as shown in Table 4 and Figure 1).

**Table 4:** Correlations among physical exercise, sensory processing sensitivity, depression-happiness, relational energy, and flourishing among vocational college students in China.

Pearson's Correlations	Pearson's r	Lower 95% CI	Upper 95% CI	Effect size (Fisher's z)	BF_10_
Level of physical activity—Level of flourishing	0.158^***^	0.140	0.176	0.159	4.349 × 10^+60^
Level of physical activity—-Sensory processing sensitivity	0.440^***^	0.425	0.454	0.472	
Level of physical activity—-Depression-happiness	0.197^***^	0.179	0.215	0.200	6.956 × 10^+95^
Level of physical activity—Relational energy	0.168^***^	0.150	0.186	0.170	8.741 × 10^+68^
Sensory processing sensitivity—Depression-happiness	0.329^***^	0.313	0.346	0.342	7.382 × 10^+281^
Sensory processing sensitivity—Level of flourishing	0.478^***^	0.464	0.492	0.521	
Sensory processing sensitivity—Relational energy	0.452^***^	0.437	0.466	0.487	
Depression-happiness—Level of flourishing	0.411^***^	0.396	0.426	0.437	
Depression-happiness—Relational energy	0.367^***^	0.351	0.383	0.385	
Level of flourishing—Relational energy	0.542^***^	0.529	0.555	0.607	

^*^P < 0.05, ^**^P < 0.01, ^***^P < 0.001. CI = confidence interval; BF_10_ = Bayes factor in favour of the alternative hypothesis.

### MIMIC and structural equation modeling (SEM) outcomes

#### Summary of MIMIC model testing: model fit and predictive path coefficients

**Figure 2:**
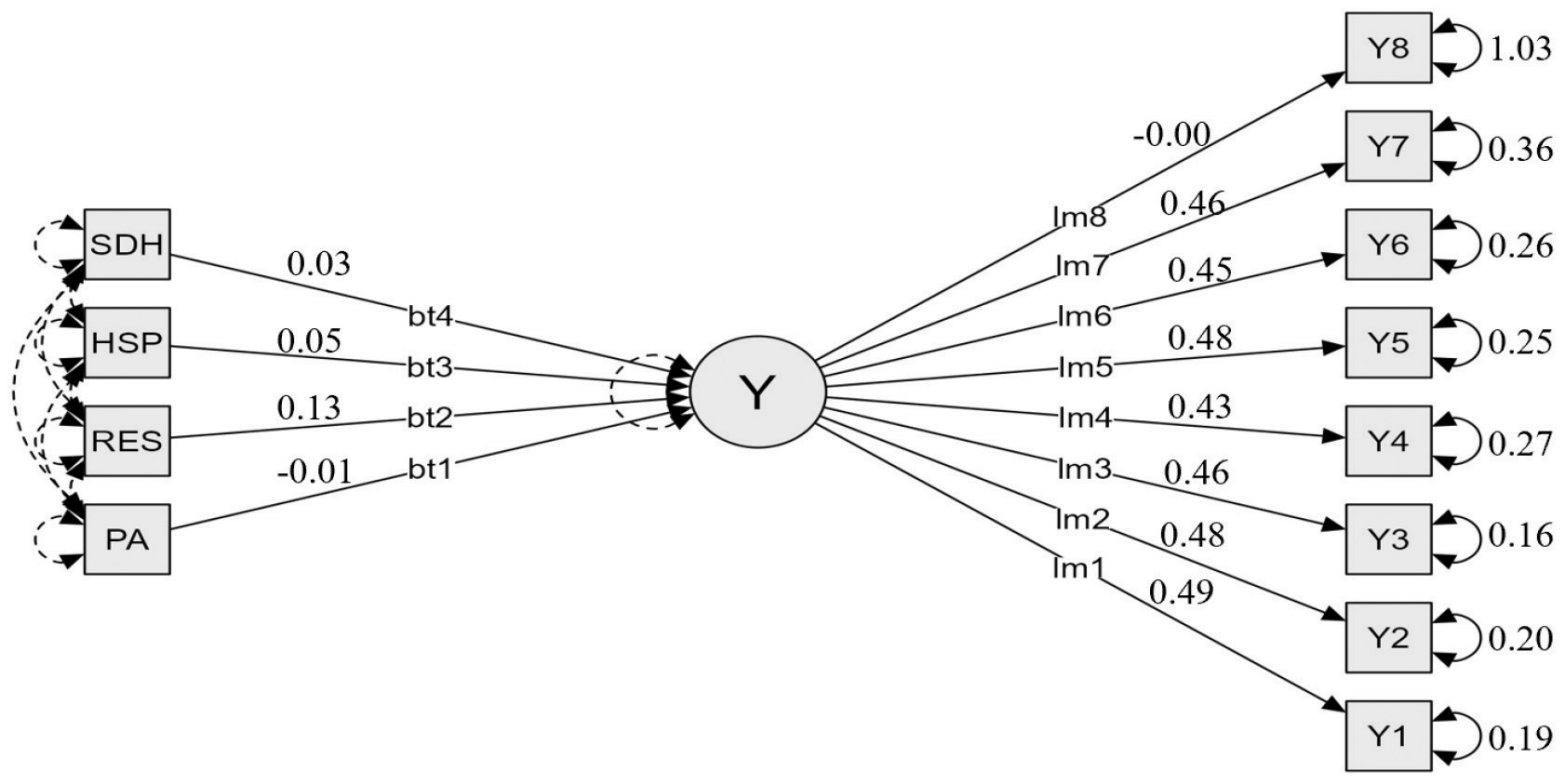
Prediction plot of the MIMIC (Multiple Indicators Multiple Causes) model for flourishing among vocational college students in China.

Random forest regression was used as an exploratory step to identify important predictors and guide SEM specification. Flourishing was modeled as a latent construct with all eight items retained as reflective indicators. In the predictor variables, relational energy (RES, β = 0.131), sensory processing sensitivity (HSPS, β = 0.046), and depression-happiness (SDHSC, β = 0.033) all had significant positive predictive effects on the latent variable (*p* < 0.001). However, physical activity level (PA) demonstrated a significant negative predictive effect (β = −0.006, *p* < 0.001). After controlling for other variables, physical exercise may affect the psychological constructs of vocational college students through complex pathways or non-linear relationships. The loadings for all observed indicators (Y1–Y7) were significant and relatively high (0.43–0.488), indicating that the measurement model has a certain level of robustness. However, the non-significant (β = −0.003, *p* = 0.662) loading of Y8 may be attributable to cultural or group-specific differences in how vocational college students interpret the statement “I am respected by others” (Item 8), as compared with the content of the other items. In conclusion, the model reveals a combined predictive mechanism for the target construct, with sensory processing sensitivity and relational energy playing key roles (as shown in Tables 5, 6 and Figure 2).

**Table 5:** Prediction test results of the MIMIC (Multiple Indicators Multiple Causes) model for flourishing among vocational college students in China.

Predictor	Estimate	Std. error	z-value	*p*	95% Confidence Interval	
					Lower bound	Upper bound
PA	−0.006	7.320 × 10^−4^	−8.516	<0.001	−0.008	−0.005
RES	0.131	0.004	36.676	<0.001	0.124	0.138
HSPS	0.046	0.001	32.484	<0.001	0.043	0.049
SDHSC	0.033	0.002	13.611	<0.001	0.028	0.038

Predictor, exogenous causal variable; estimate, unstandardized regression coefficient; Std. error, standard error; Z-value, z-statistic; P, statistical significance value; 95% Confidence Interval, 95% confidence interval.

**Table 6:** Path coefficients of the MIMIC (Multiple Indicators Multiple Causes) model for flourishing among vocational college students in China.

Indicator	Estimate	Std. error	z-value	*p*	95% Confidence Interval	
					Lower bound	Upper bound
Y1	0.488	0.005	101.351	<0.001	0.479	0.498
Y2	0.476	0.005	98.65	<0.001	0.466	0.485
Y3	0.46	0.004	102.714	<0.001	0.451	0.469
Y4	0.43	0.005	85.524	<0.001	0.42	0.44
Y5	0.481	0.005	93.599	<0.001	0.471	0.492
Y6	0.453	0.005	89.009	<0.001	0.443	0.463
Y7	0.455	0.006	80.764	<0.001	0.444	0.466
Y8	−0.003	0.008	−0.438	0.662	−0.019	0.012

Predictor, exogenous causal variable; Estimate, unstandardized regression coefficient; Std. error, standard error; Z-value, z-statistic; *P*, statistical significance value; 95% Confidence Interval, 95% confidence interval.

#### Multiple linear regression analysis

Model 1 showed that BMI, gender, and grade explained little variance in flourishing, with only BMI emerging as a significant predictor. After adding relational energy, sensory processing sensitivity, depression–happiness, and physical exercise, Model 2 showed a substantial improvement in explanatory power, accounting for 39.8% of the variance in flourishing. Relational energy, sensory processing sensitivity, and depression–happiness were significant positive predictors, whereas physical exercise showed a small negative effect, possibly due to overlap with other predictors and a suppressor effect (as shown in Table 7).

**Table 7:** Linear regression analysis of factors associated with flourishing among vocational college students in China.

Variable	Model 1		Model 2	
	β	*t*	β	*t*
BMI	0.185	18.886^***^	−0.007	−0.63
Gender	0.007	0.754	−0.006	−0.831
Grade	0.003	0.349	−0.012	−1.642
RES			0.352	41.625^***^
HSPS			0.282	30.873^***^
SDHSC			0.202	25.268^***^
PA			−0.057	−5.37^***^
Adjusted R-squared	0.035		0.398	
*F*	138.438^***^		1,076.74^***^	

^*^P < 0.05, ^**^P < 0.01, ^***^P < 0.001. β = standardized regression coefficient; *t* = t statistic. Adjusted R-squared, adjusted coefficient of determination; F-value, F-statistic for testing the overall significance of the model.

#### Comprehensive predictive analysis and evaluation of the MIMIC model

**Figure 3:**
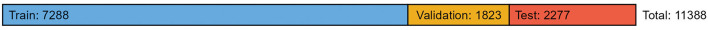
Training and testing data split for the random forest regression analysis.

**Figure 4:**
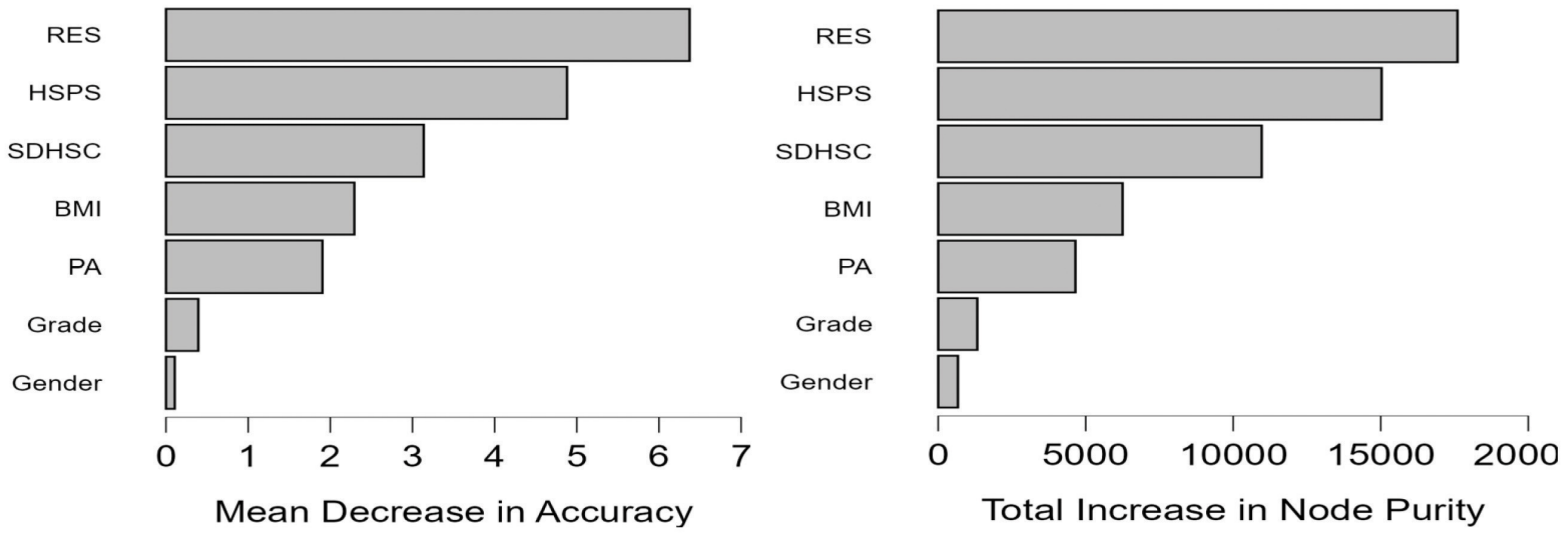
Bar chart of feature importance in the random forest regression model predicting flourishing among vocational college students in China.

**Figure 5:**
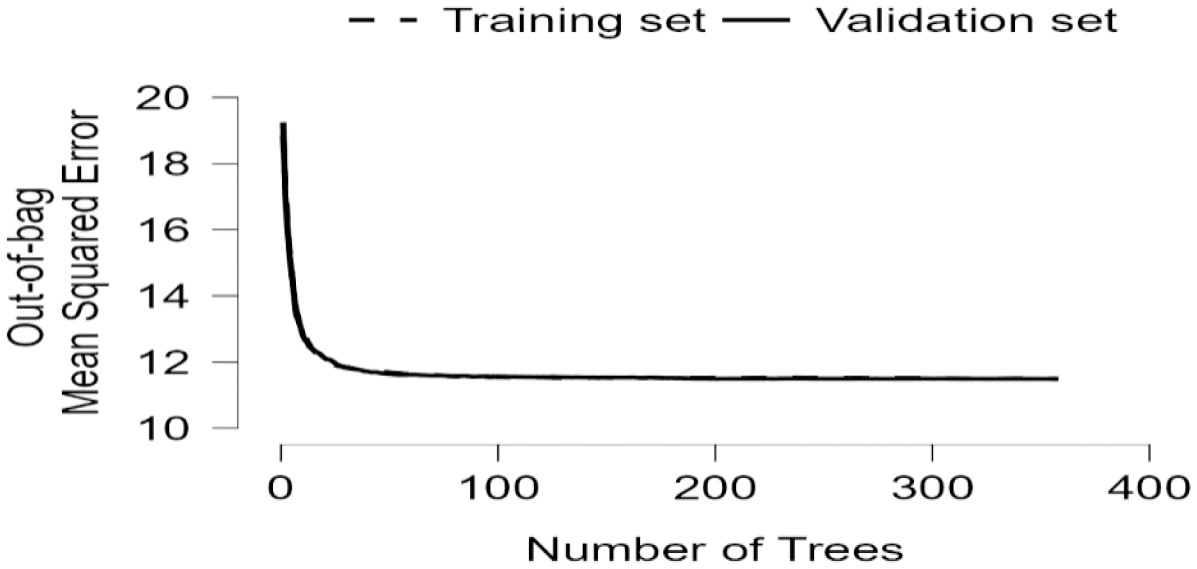
Out-of-bag mean squared error plot for the random forest regression model.

**Figure 6:**
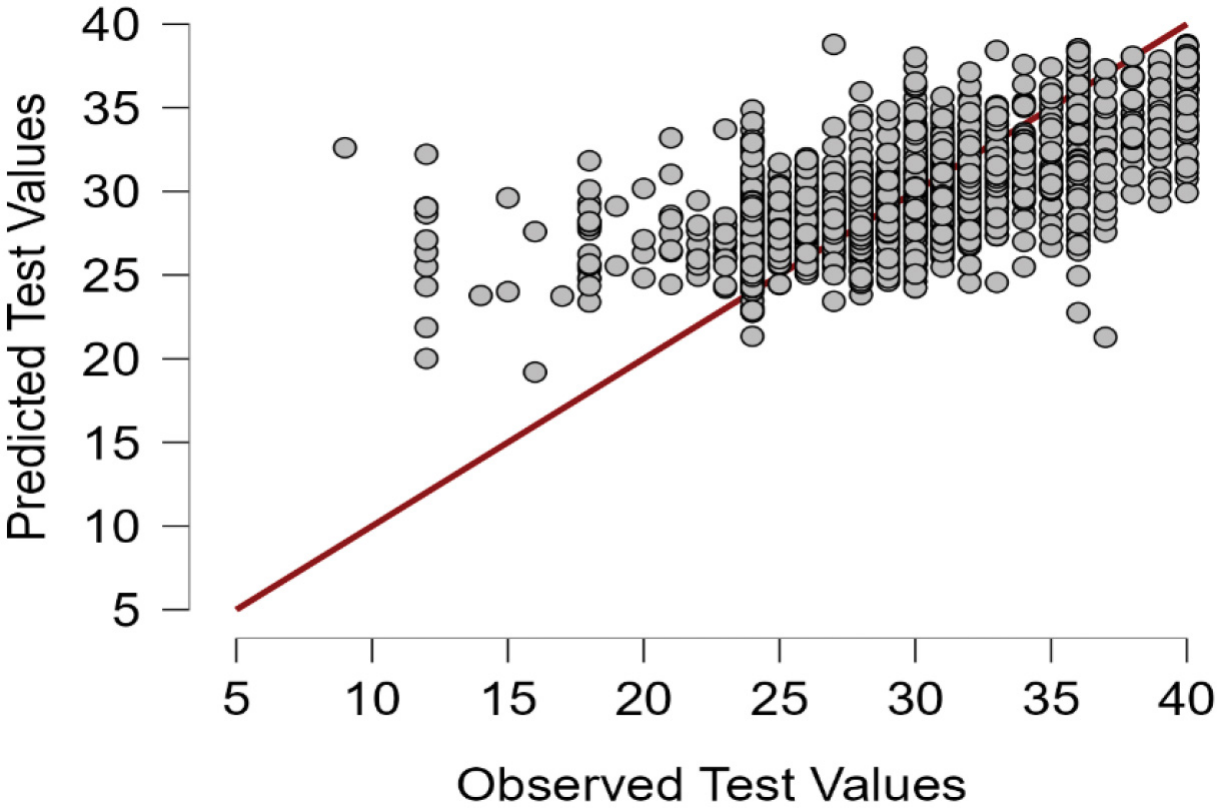
Predictive performance of the random forest regression model for flourishing among vocational college students in China.

RFR can go beyond the limitations of traditional linear models and help identify potential non-linear dynamics among variables, thereby supporting the robustness of the selected predictors. The random forest model showed good stability and generalisability, with similar error values across the validation, test, and out-of-bag estimates. Feature importance analysis indicated that relational energy, sensory processing sensitivity, and depression–happiness were the strongest predictors, followed by BMI and physical activity, whereas grade and gender contributed relatively little (as shown in Tables 8–10; Figures 3–6).

**Table 8:** Random forest regression results for predicting flourishing among vocational college students in China.

Trees	Features per split	*n*(Train)	*n*(Validation)	*n*(Test)	Validation MSE	Test MSE	OOB Error
358	2	7,288	1,823	2,277	11.87	11.32	11.48

Trees, number of decision trees; features per split, number of features randomly selected at each split; *n*(train), training set size; *n*(validation), validation set size; *n*(test), test set size; validation MSE, mean squared error of the validation set; test MSE, mean squared error of the test set; OOB error, out-of-bag error.

**Table 9:** Model performance metrics of the random forest regression model for flourishing among vocational college students in China.

Performance metric	Values
MSE	11.37
MSE (scaled)	0.717
RMSE	3.372
MAE/MAD	2.463
MAPE	9.06 %
*R^2^*	0.411

MSE, mean squared error; MSE (scaled), standardized mean squared error; RMSE, root mean squared error; MAE/MAD, mean absolute error / mean absolute deviation; MAPE, mean absolute percentage error.

**Table 10:** Feature importance metrics from the random forest regression model for flourishing among vocational college students in China.

Variable	Mean decrease in accuracy	Total increase in node purity	Mean dropout loss
RES	6.373	1,7601.70	3.818
HSPS	4.881	1,5029.50	3.592
SDHSC	3.137	1,0965.80	3.387
BMI	2.293	6,250.50	3.014
PA	1.904	4,651.60	2.959
Grade	0.393	1,325.40	2.791
Gender	0.107	669.1	2.683

Mean Decrease in Accuracy, mean decrease in accuracy; Total Increase in Node Purity, total increase in node purity; Mean Dropout Loss, mean dropout loss.

### Moderated chain mediation effect test

**Figure 7:**
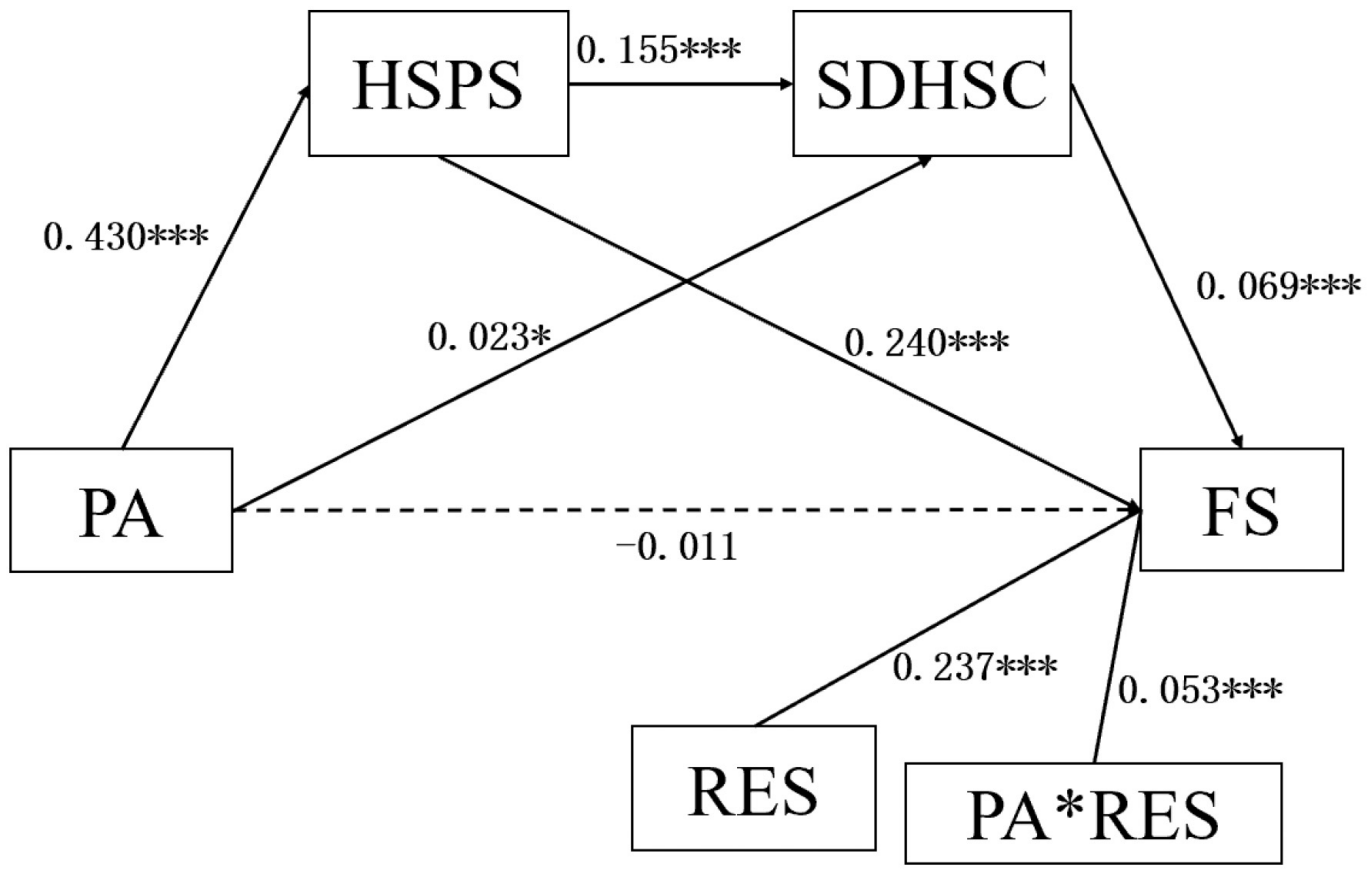
Moderated chain mediation model linking physical exercise to flourishing among vocational college students in China. ^*^P < 0.05, ^***^P < 0.001.

The moderated chain mediation model showed that physical exercise did not directly predict flourishing, but it positively predicted sensory processing sensitivity, which in turn contributed to the chain pathway to flourishing through depression–happiness. In addition, relational energy significantly moderated the association between depression–happiness and flourishing. These results support the proposed moderated chain mediation model (as shown in Figure 7).

The 95% confidence intervals showed that all three indirect effects were significant, as none of the intervals included zero. This indicates that the mediation pathways were stable across different levels of physical exercise, supporting the moderating role of physical exercise in the model. Therefore, H2 and H3 were supported (as shown in Table 11).

**Table 11:** Bootstrap 95% confidence intervals and indirect effects in the moderated chain mediation model among vocational college students in China.

Path	Moderator	Effect size	Standard error	95% Confidence Interval	
				Lower limit	Upper limit
PA → HSPS → FS	——	0.0355	0.0018	0.032	0.0391
PA → SDHSC → FS	*M-1SD*	0.0033	0.0006	0.0021	0.0046
	*M*	0.0037	0.0006	0.0024	0.005
	*M+1SD*	0.0041	0.0007	0.0027	0.0055
PA → HSPS → SDHSC → FS	*M-1SD*	0.0067	0.0006	0.0055	0.008
	*M*	0.0075	0.0005	0.0065	0.0086
	*M+1SD*	0.0083	0.0006	0.0072	0.0094

**Figure 8:**
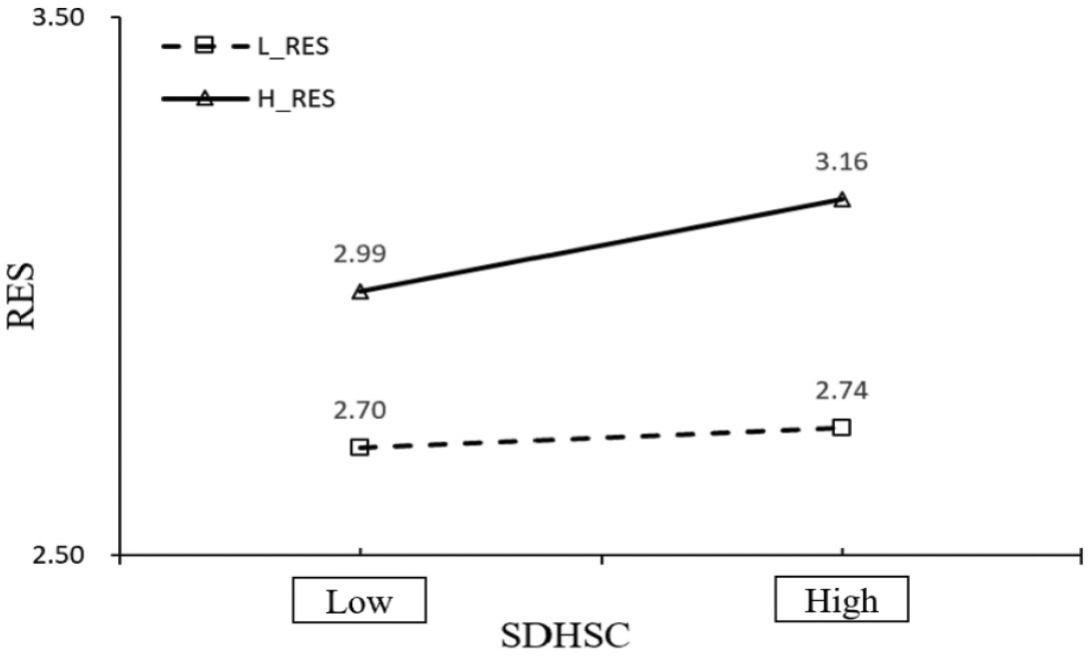
Simple slope plot of the moderating effect of relational energy on the association between depression-happiness and flourishing.

The simple slope analysis showed that relational energy significantly strengthened the positive association between depression–happiness and flourishing. Although this relationship was significant at both low and high levels of relational energy, it was stronger when relational energy was high, supporting H4 (as shown in Figure 8).

### Robustness test of the model

**Figure 9:**
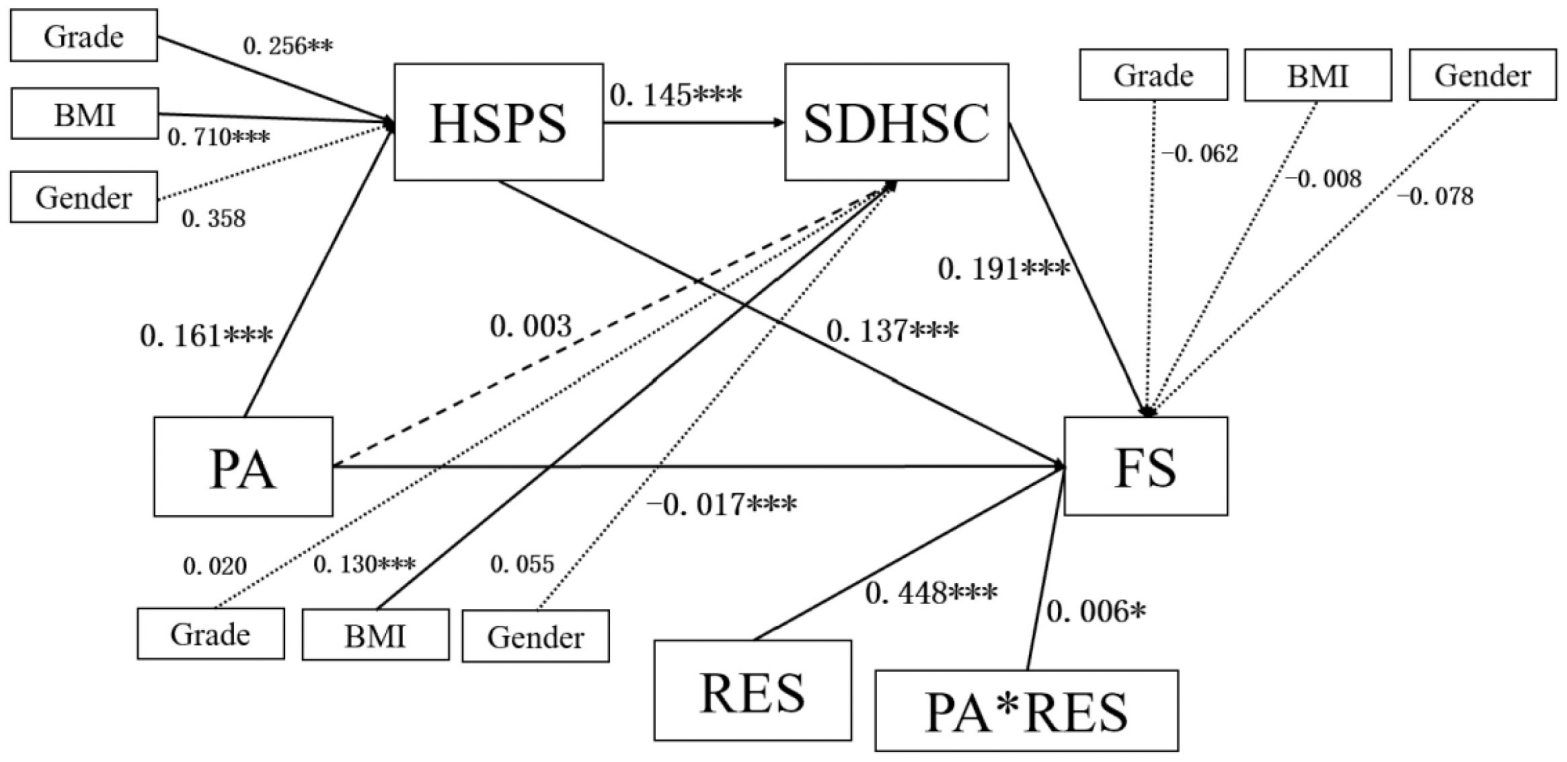
Moderated chain mediation model with control variables among vocational college students in China. ^*^P < 0.05, ^**^P < 0.01, ^***^P < 0.001.

After controlling for gender, grade, and BMI, the moderated chain mediation path remained significant, although the coefficients were slightly reduced, indicating that the model was robust. The control variables partly explained the indirect effect of physical exercise on flourishing through sensory processing sensitivity and depression–happiness. Notably, physical exercise showed a significant negative direct effect on flourishing after adjustment, suggesting a possible suppressor effect or context-dependent relationship (as shown in Figure 9).

Moreover, the interaction between physical exercise and relational energy remains significant, suggesting that the psychological resilience of vocational college students can buffer the potential negative effects of physical exercise. Therefore, the focus should not only be on “more exercise,” but also on “how to exercise” and “for whom to exercise.”

## Discussion

The study suggests that the direct effect of physical exercise on flourishing levels is not significant, but it produces a positive indirect effect through the mediating chain “sensory processing sensitivity → depression-happiness.” Relational energy may strengthen the association between depression, happiness, and flourishing, suggesting it could play a moderating role in the relationship between emotional states and flourishing.

### Physical exercise and flourishing: an indirect rather than direct association

A central finding of this study is that physical exercise did not show a significant direct association with flourishing in the primary model but did so indirectly through the proposed mediating variables. This is broadly consistent with previous research showing that the mental health benefits of physical exercise are often explained through intermediate mechanisms, such as emotional regulation, self-esteem, resilience, or social support, rather than through a single direct pathway (45). In this sense, the present findings support the growing view that exercise contributes to psychological wellbeing through internal transformation processes. At the same time, our results extend previous work by identifying a more specific pathway involving SPS and depression-happiness. This suggests that, among vocational college students, exercise may be related to flourishing not merely because students are physically active, but because activity may be associated with how they process internal and external experiences and how these experiences are reflected in their emotional functioning (46). This interpretation may help explain why increases in physical activity do not necessarily yield uniform psychological benefits across student populations. In particular, interventions that focus primarily on exercise frequency or duration, while overlooking motivational quality, emotional regulation, and broader psychosocial processes, may produce limited or inconsistent effects on psychological wellbeing (47).

### The mediating role of sensory processing sensitivity

Another important finding is that SPS served as the first mediating variable in the model. This result suggests that physical exercise may be associated with flourishing, at least in part, through individual differences in sensitivity and perceptual processing. Although SPS is traditionally treated as a relatively stable trait, recent literature has increasingly suggested that environmental experiences may shape how sensitivity is expressed and managed (46). In the present study, higher levels of physical exercise were positively associated with SPS, which in turn was related to depression, happiness, and flourishing. This finding may be understood in two ways. On the one hand, regular exercise may provide repeated bodily and sensory input that supports interoceptive processing; over time, this may help students become more aware of internal states and better able to interpret them (48). On the other hand, exercise may offer a structured and controllable context in which sensitive individuals can regulate arousal and respond adaptively to stimulation (49). Therefore, rather than viewing sensitivity only as a vulnerability factor, the present findings are more in line with perspectives that regard sensitivity as a characteristic that may confer advantages under supportive conditions (50, 51). Compared with previous studies linking SPS to affective outcomes and wellbeing, the current study provides evidence that SPS may also be relevant to exercise-related mental health processes. This has practical implications. For student mental health promotion, exercise programmes may be more effective when they are not “one-size-fits-all,” but instead provide adjustable intensity, enjoyable formats, and psychologically safe environments, especially for students who are more sensitive to stimulation.

### Depression–happiness as an emotional link to flourishing

The results also showed that depression–happiness was positively associated with flourishing and functioned as a second mediator in the chain. This finding highlights the importance of emotional functioning within the dual-factor model of mental health, which argues that mental health is not merely the absence of symptoms but also the presence of positive functioning (52, 53). In this study, flourishing was more likely to be observed among students with more favorable emotional functioning, consistent with the view that positive mental health involves not only the absence or reduction of symptoms but also the presence of emotional, psychological, and social wellbeing (54). Although the positive association between depression, happiness, and flourishing may initially appear unexpected, it is likely to reflect the scale's scoring characteristics and the non-clinical nature of the sample. Therefore, the current findings do not necessarily contradict prior studies; rather, they reinforce the broader literature suggesting that emotional wellbeing and flourishing are closely interconnected. However, the present findings suggest that improving flourishing may require a broader mental health approach that includes both symptom-related support and the active cultivation of positive functioning, meaning, optimism, and emotional balance. For colleges and vocational institutions, this may justify integrating physical activity initiatives with counseling, psychoeducation, and emotional skills training.

### The moderating role of relational energy

The moderating effect of relational energy is another key contribution of this study. Specifically, relational energy strengthened the positive association between depression, happiness, and flourishing. This suggests that even when students have relatively favorable emotional functioning, the degree to which this translates into flourishing may depend partly on their interpersonal environment. Students who receive vitality, encouragement, and emotional support from others may be better able to convert short-term positive emotional states into more stable psychological resources, such as meaning, self-development, and life satisfaction (55, 56). This finding is consistent with resource-based perspectives, which view social support as a key interpersonal resource, and with prior research showing that social connectedness and supportive relationships are important foundations of wellbeing (57). It also adds nuance to the literature by showing that social resources may not simply have a direct effect on flourishing but also function as boundary conditions that shape how emotional experiences influence positive outcomes. From a practical perspective, this result suggests that exercise-based mental health promotion should not be designed solely as an individual-level behavioral intervention. Its benefits may be enhanced when implemented in relationally supportive contexts, such as peer-based exercise groups, encouraging teacher-student interactions, or campus environments that foster belonging and emotional vitality.

### A more cautious interpretation of the negative direct association after controlling for covariates

In the robustness analysis, after controlling for gender, grade, and BMI, the direct association between physical exercise and flourishing became significantly negative. This pattern should be interpreted cautiously. Rather than concluding that physical exercise harms flourishing, the result is more likely to indicate that the relationship is conditional, context-dependent, and shaped by competing mechanisms. Once demographic and body-related factors were statistically controlled, the model may have revealed a residual association that reflects stress, burden, compulsive exercise tendencies, or other unmeasured pressures linked to exercise participation in some students (20, 58). This interpretation contrasts with studies reporting a generally positive association between physical activity and wellbeing. Still, it is not inconsistent with evidence showing that exercise is not universally beneficial under all circumstances. Previous research has suggested that when exercise becomes externally pressured, excessive, or linked to performance anxiety, body image concerns, or stress, its psychological value may weaken or even reverse (59). Therefore, the present study supports a more nuanced understanding: physical exercise is not inherently beneficial in all forms, and its association with flourishing depends on how it is experienced, regulated, and socially supported. This point is particularly relevant for current educational practice. Institutions should avoid promoting exercise solely through quantitative targets or mandatory participation. Instead, policies should emphasize enjoyment, autonomy, manageable intensity, and supportive delivery. In other words, “more exercise” should not automatically be treated as the same as “better mental health” (60).

## Limitation

Despite the insights gained from this large-scale investigation into the mental health mechanisms of vocational college students, several limitations remain. Firstly, regarding measurement, the data rely primarily on self-report scales. Albeit common method bias (CMB) was found to be within an acceptable range, the reliance on self-assessment cannot entirely preclude social desirability bias or subjective cognitive distortion. Future research should consider incorporating multi-source evaluations or physiological indices (e.g., cortisol levels and heart rate variability) to enhance data objectivity. Secondly, constrained by the cross-sectional design, this study is unable to establish rigorous causal sequencing over time, necessitating longitudinal tracking in future studies to further validate the model's stability. Finally, comparative studies across different cultures and cohorts should be conducted to verify the generalizability and boundary conditions of the proposed model.

## Conclusions

Among vocational college students, the relationship between physical exercise and flourishing is complex and involves multiple psychological and social variables. Significant associations were found among physical exercise, sensory processing sensitivity, depression-happiness, relational energy, and flourishing. After controlling for factors such as gender, grade, and body mass index, physical exercise showed a significant negative direct association with flourishing. In future physical and health education in vocational colleges, the emphasis should not be placed solely on the “dose” of exercise, such as frequency and intensity, but also on the “quality” of exercise and its fit with individual psychological characteristics. Through teamwork and teacher–student interaction, what might otherwise remain mere physical exertion should be transformed into a setting for positive emotional experience.

## Data Availability

The original contributions presented in the study are included in the article/supplementary material, further inquiries can be directed to the corresponding author.
